# A Secreted Form of the Asialoglycoprotein Receptor, sH2a, as a Novel Potential Noninvasive Marker for Liver Fibrosis

**DOI:** 10.1371/journal.pone.0027210

**Published:** 2011-11-11

**Authors:** Elena Veselkin, Maria Kondratyev, Yoav Lurie, Efrat Ron, Moshe Santo, Shimon Reif, Irma Elashvili, Lana Bar, Gerardo Z. Lederkremer

**Affiliations:** 1 Department of Cell Research and Immunology, George Wise Faculty of Life Sciences, Tel Aviv University, Tel Aviv, Israel; 2 Liver Unit, Department of Gastroenterology, Tel Aviv Sourasky Medical Center, Tel Aviv, Israel; Institute of Molecular and Cell Biology, Singapore

## Abstract

**Background and Aim:**

The human asialoglycoprotein receptor is a membrane heterooligomer expressed exclusively in hepatocytes. A soluble secreted form, sH2a, arises, not by shedding at the cell surface, but by intracellular cleavage of its membrane-bound precursor, which is encoded by an alternatively spliced form of the receptor H2 subunit. Here we determined and report that sH2a, present at constant levels in serum from healthy individuals is altered upon liver fibrosis, reflecting the status of hepatocyte function.

**Methods:**

We measured sH2a levels in serum using a monoclonal antibody and an ELISA assay that we developed, comparing with routine liver function markers. We compared blindly pretreatment serum samples from a cohort of 44 hepatitis C patients, which had METAVIR-scored biopsies, with 28 healthy individuals.

**Results:**

sH2a levels varied minimally for the healthy individuals (150±21 ng/ml), whereas the levels deviated from this normal range increasingly in correlation with fibrosis stage. A simple algorithm combining sH2a levels with those of alanine aminotransferase allowed prediction of fibrosis stage, with a very high area under the ROC curve of 0.86.

**Conclusions:**

sH2a has the potential to be a uniquely sensitive and specific novel marker for liver fibrosis and function.

## Introduction

A soluble secreted form of the human asialoglycoprotein receptor (ASGPR), termed sH2a, is formed by cleavage in the endoplasmic reticulum of hepatocytes of its precursor [1], encoded by an alternatively spliced variant of the ASGPR H2 subunit mRNA [2]. We recently reported that sH2a is present at constant levels in the serum of healthy individuals [3]. The ASGPR is expressed to a significant degree only in hepatocytes [4] and serves in the clearance of asialoglycoproteins from the plasma [5]. Several conditions correlate with a significant reduction in the levels of the ASGPR, among them hepatocyte dedifferentiation [6], [7], chronic alcohol consumption [8] and liver fibrosis and cirrhosis [9], [10]. We found that the levels of sH2a are also significantly reduced in a pilot evaluation of HCV patients with cirrhosis [3]. Therefore, we reasoned that sH2a might be regulated in a similar fashion as the membrane ASGPR and could constitute a unique non-invasive marker for hepatocyte function and fibrosis. Such a marker is presently lacking, and despite proposed experimental markers or combinations of routine tests, the gold standard continues to be biopsy [11], [12]. Besides its invasiveness and concomitant risks, biopsy is not highly accurate in predicting fibrosis stage, because of the sampling error due to the small volume of the samples and inter-observer variation of up to 20% [13].

Experimental serum markers that have been proposed for fibrosis include extracellular matrix (ECM) macromolecules and their degradation products. Unfortunately, these markers are not very sensitive and are not liver specific, they can also reflect inflammatory processes in other tissues. The same is true for the levels of normally intracellular enzymes (alanine aminotransferase (ALT), aspartate aminotransferase (AST), γ-glutamyl transpeptidase (GGT), etc.). These markers are indicative of liver damage but do not reflect directly hepatocyte function. Function is diagnosed with classical markers like albumin and prothrombin time (PT), but these are sensitive only to severe disease. Likewise, methods that combine several markers, like Fibrotest [14], are suitable to detect cirrhosis and advanced fibrosis, but are not satisfactory for moderate fibrosis.

Therefore, it would be highly beneficial to have a non-invasive serum marker that is a specific and sensitive test for hepatocyte function, which would also serve as an early indicator of fibrosis. Here we show for a cohort of HCV patients compared to healthy individuals that ASGPR sH2a may be a valid candidate for such a role.

## Materials and Methods

### Ethics Statement

The study had a priori approval by Tel Aviv Sourasky Medical Center hospital ethical committee according to the Helsinki Declaration and written informed consent was obtained from all participants.

### Materials, antibodies and ELISA

Alkaline phosphatase (ALP) substrate p-nitrophenylphosphate (p-NPP) was from Chemicon International (Temecula, CA). Imperial protein stain was from (Pierce). Common reagents were from Sigma.

The preparation of a monoclonal antibody against sH2a and the development of a competitive ELISA using this antibody were described before [3]. ALP-conjugated goat anti-mouse IgG antibody was from Jackson Laboratories (West Grove, PA).

### Study subjects

Retrospective samples were from a group of healthy blood donors and a cohort of consecutive pretreatment HCV-infected patients at the Liver Unit, Tel Aviv Sourasky Medical Center. Patients co-infected with HIV, HBV or with additional diseases from other etiologies were excluded. Overweight healthy individuals were also excluded.

### Routine laboratory tests

Patients had routine laboratory tests performed by a certified central lab, using common commercial methods. These tests included total bilirubin, ALP, ALT, AST, GGT, albumin, serum cholesterol, PT and HCV RNA level (RT-PCR). Similar tests were done on healthy individuals except for PT and HCV RNA.

### Liver histology

Percutaneous liver biopsy was performed using a Tru-Core II [R] biopsy instrument under ultrasound guidance. A single pathologist blinded to all clinical and serological results evaluated all slides. Two patients where biopsies were found inadequate were excluded from the study. Biopsies were METAVIR-scored [15] for fibrosis stage and inflammation grade.

### Statistical analysis

Comparison between groups of patients with regard to demographic (age, gender) and clinical parameters was performed using Chi-square tests and Kruskal-Wallis non-parametric analysis of variance (ANOVA), as applicable. The association between stage and other parameters was examined using logistic regression. Results are presented as odds ratio (OR), sensitivity and specificity with 95% confidence intervals (CI). The diagnostic value of the combination score of sH2a and ALT with regard to fibrosis stages was evaluated by the area under the receiver operating characteristic curve (AUROC). The statistical significance level was set to 0.05. SPSS for Windows software, version 14.0 (Chicago, IL) was used for the analysis.

## Results

### Comparison of the serum levels of sH2a in a cohort of HCV patients with those in healthy individuals

We recently showed that sH2a is present at constant levels in healthy individuals and strongly reduced in cirrhotic HCV patients [3]. To investigate whether sH2a levels correlate with fibrosis, we analyzed blindly and randomly, using an ELISA test that we had developed, samples of serum obtained from 44 pretreatment HCV patients and from 28 healthy individuals. Both populations had male and female individuals with a similar wide range of ages (Tables 1 and 2). HCV patients were all ambulatory and minimally symptomatic. HCV patients had biopsies taken that gave a median METAVIR- fibrosis stage of 1 and ranges 0–4. The samples were also analyzed for a series of routine liver tests, and the HCV patients showed significantly higher median levels than the healthy individuals for bilirubin, ALP, ALT, AST, GGT but only the median levels of ALT and AST were above the normal, other median levels including those of albumin and PT were in the normal range for both populations. The levels of sH2a were quite constant for the healthy group, 150±21 ng/ml, giving a median of 148 ng/ml, with a range of 105–188 ng/ml (interquartile range (IQR): 136–163 ng/ml) (Table 2). For the HCV patients the sH2a median was similar, 145 ng/ml, but the range was much wider, 31–237 ng/ml (Table 1).

**Table 1:** HCV patients.

Characteristic	Median	[IQR]	range
Male gender (%)	63.64		
Age	41.5	29–53	19–64
Weight (kg)	73	66–84	56–127
total bilirubin (mg/dl)	2	1–3	0.3–4
bilirubin/normal^1^	0.29	0.14–0.43	0.04–0.71
ALP (U/l)	75	62–96.5	44–194
ALP/normal^1^	0.63	0.50–0.77	0.36–1.50
ALT (U/l)	75	43–103	30–458
ALT/normal^1^	1.77	1.21–2.66	0.70–13.47
AST(U/l)	47	33–59	21–291
AST/normal^1^	1.31	0.96–1.67	0.58–8.56
GGT (U/l)	33	23–52	10–246
GGT/normal^1^	0.56	0.37–0.94	0.18–5.02
albumin (g/l)	45	43–47	38–54
albumin/normal^1^	0.92	0.88–0.96	0.78–1.10
serum cholesterol (mg/dl)	166	142–199	102–323
cholesterol/normal^1^	0.83	0.71–1.00	0.51–1.62
PT (sec)	12	11.4–12.2	10.4–14.8
PT/normal^1^	0.81	0.77–0.82	0.70–0.99
sH2a (ng/ml)	145	118–167	31–237
Fibrosis stage	1	0–3	0–4
Inflammation grade	2	1–3	0–4

1 Median of absolute values divided by upper limit of normal range.

**Table 2:** Healthy individuals.

Characteristic	Median	[IQR]	range
Male gender (%)	53.57		
Age	40.00	29–48	22–64
total bilirubin (mg/dl)	0.36	0.22–0.50	0.06–1.05
bilirubin/normal	0.05	0.05–0.16	0.009–0.42
ALP (U/l)	48.00	36–55	22–88
ALP/normal	0.35	0.33–0.51	0.17–0.68
ALT (U/l)	19.00	14–27	9–38
ALT/normal	0.42	0.37–0.65	0.26–1.03
AST(U/l)	27.00	20–31	14–39
AST/normal	0.67	0.49–0.89	0.35–1.08
GGT (U/l)	12.50	3–18	1–38
GGT/normal	0.18	0.06–0.38	0.02–0.62
albumin (g/l)	43.00	41–46	31–50
albumin/normal	1.24	1.20–1.39	0.89–1.52
serum cholesterol (mg/dl)	190.00	167–219	101–274
cholesterol/normal	0.86	0.83–1.09	0.50–1.37
sH2a (ng/ml)	148	136–163	105–188

**Figure 1:**
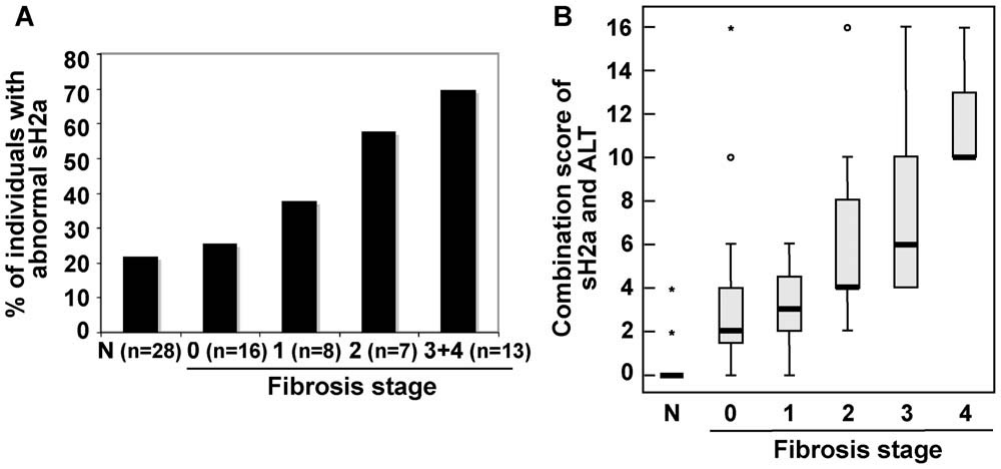
Box plots of sH2a and of the combination score of sH2a and ALT versus fibrosis stage. **A**) The levels of sH2a in sera were compared between a group of 28 healthy individuals (N) and 44 HCV patients with different METAVIR fibrosis stages (0–4). Plotted is the percent of individuals in each group that showed abnormal sH2a levels (<125 ng/ml or > = 175 ng/ml). In parenthesis are the numbers of individuals in each group. P value (Chi-square tests, comparing stages 3+4 to 0) = 0.034. **B**) The serum levels of sH2a and ALT for each healthy individual and for each HCV patient were joined in a combined score. Shown is a boxplot of the combination score of sH2a and ALT for healthy individuals (N) and for HCV patients grouped by METAVIR fibrosis stage (0–4). The box height indicates the IQR in each group. The thick horizontal bar is the median. The whiskers extend to the farthest non-outlier value smaller than 1.5×IQR. Circles indicate “mild” outliers (<3×IQR) and asterisks show “extreme” outliers (>3×IQR). P<0.0001. The combination score of sH2a and ALT was calculated as follows: Combination score = (1 if normalized ALT<1.1, 2 if normalized ALT 1.1–2, 3 otherwise)×(2 if sH2a 125–175 ng/ml, 4 if sH2a 113–124 ng/ml or 176–207 ng/ml, 6 otherwise)-2.

We analyzed the relation between levels of the different markers and fibrosis stage (Table 3). There was a moderate increase in the likelihood for very high levels of ALT with increasing fibrosis stage. There was a significant increase in the likelihood for high levels of ALP although the percent of patients at fibrosis stages 3–4 with high ALP was still low (23%). There was a stronger correlation between the levels of AST and GGT with fibrosis stage, but few patients showed abnormal GGT and therefore its correlation was not statistically significant. There was also a strong correlation of likelihood for abnormal sH2a levels with increasing fibrosis stage. This abnormal sH2a range was taken arbitrarily as <125 ng/ml or > = 175 ng/ml, starting about 12 ng/ml below and above the IQR of the healthy group. Most individuals with abnormal sH2a had a decreased level of the marker in serum, but a few (7) presented an abnormally high level. These few cases might reflect the start of a neoplastic process as discussed later. There was considerable difference in the percent of individuals with abnormal sH2a between the group with mild or moderate fibrosis (stages 0–2) (35.5%) to those with advanced fibrosis (stages 3–4) (69.2%) (Table 3). As shown in Fig. 1A, there was a steady increase in the number of individuals with an abnormal level of sH2a with increasing fibrosis stage. All patients at stage 4 showed abnormal sH2a levels, but because of the small number of samples at this stage they were joined with those at stage 3 to achieve statistical significance (Fig. 1A). Most noteworthy, sH2a shows a change to abnormal levels in a significant portion of patients at mild and intermediate fibrosis stages (1–2).

**Table 3:** Univariate analysis of correlation of characteristics with advanced fibrosis.

Characteristic	Healthy	HCV patients		P value	Odds ratio (95% CI)
		Fibrosis stage			
	N (n = 28)	0–2 (n = 31)	3–4 (n = 13)		
Male gender^1^	15 (53.6)	18 (58.1)	10 (76.9)	0.235	2.41 (0.55–10.52)
Median age (IQR)	40 (29–48)	35 (27–49)	51 (47–55)	0.019	1.08 (1.01–1.16)
bilirubin/normal >1	0 (0)	0 (0)	0 (0)	-	-
ALP/normal >1	0 (0)	1 (3.23)	3 (23.1)	0.027	10.00 (0.92–108.33)
ALT/normal >2	0 (0)	10 (32.3)	8 (61.5)	0.047	4.20 (1.09–17.32)
AST/normal >1	1 (3.6)	18 (58.1)	12 (92.3)	0.008	9.33 (1.81–48.24)
GGT/normal >1	0 (0)	5 (16.1)	5 (38.5)	0.075	3.71 (0.83–16.55)
albumin/normal <0.8	0 (0)	1 (3.23)	0 (0)	0.529	1.4 (1.16–1.70)
cholesterol/normal >1.2	2 (7.1)	1 (3.23)	1 (7.69)	0.476	2.73 (0.16–47.46)
abnormal sH2a	6 (21.4)	11 (35.5)	9 (69.2)	0.040	4.09 (1.02–16.40)
(sH2a <125 ng/ml or > = 175 ng/ml)					

1 Data is presented as n (%) except for age, which is presented as median (IQR); odds ratios are from simple logistic regression, using the forced entry method, on each variable. The dependent variable in the regression is state and the outcome is METAVIR score (1 if state is 3–4); CI, confidence interval presented for each explanatory variable.

### A combined score of the levels of sH2a and ALT

**Figure 2:**
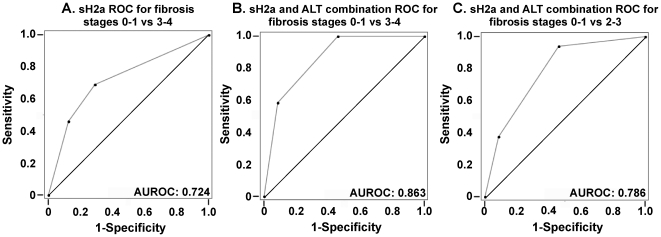
ROC plots of sH2a and of the combination score of sH2a and ALT. **A**) Receiver operating characteristic (ROC) plot of sH2a values for advanced fibrosis and cirrhosis (METAVIR 0–1 vs 3–4) for the group of 44 HCV patients. **B–C**) ROC plots of the combined scores of sH2a and ALT were made for advanced fibrosis and cirrhosis (METAVIR 0–1 vs 3–4) (B) or for intermediate fibrosis (METAVIR 0–1 vs 2–3) (C) of the same group of patients.

Given that sH2a levels would reflect liver function, whereas the levels of ALT are indicative of liver damage, we reasoned that a combination of these values would compensate for individual fluctuations in the overall state of each patient. An algorithm combining the levels of sH2a with those of ALT, gave a score of zero for the healthy individuals and a very good correlation with fibrosis stage for the HCV patients (Fig. 1B). Details of the calculation of the combined value are given in the legend to Fig. 1B. Using this combined score of sH2a and ALT levels, the AUROC calculated for advanced fibrosis (stages 3–4) was very high, 0.863 (Fig. 2B), compared to that of sH2a alone (0.724, Fig. 2A). The AUROC was also high for this combined value, 0.786, when calculated for intermediate fibrosis (stages 2–3) (Fig. 2C). For intermediate and significant fibrosis (stages 2–4), at a sensitivity of 65%, the specificity of sH2a and ALT was similar, 71% and 73% respectively, but the accuracy of sH2a was higher, 68% versus 58% for ALT. At the same sensitivity the combination score gave a high specificity, 85%.

## Discussion

**Figure 3:**
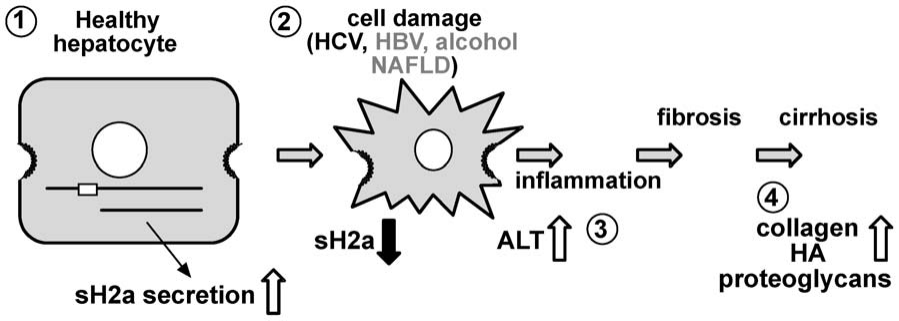
Model depicting the sequence of events in the fibrogenic process and changes in sH2a secretion. The membrane-bound precursor of sH2a is cleaved in the endoplasmic reticulum of healthy hepatocytes, traverses the secretory pathway and is secreted to the plasma (step 1). As a consequence of HCV infection and possibly also upon other insults, hepatocytes are damaged, their overall function is compromised and sH2a secretion is reduced, while intracellular enzymes like ALT are released to the plasma (step 2). This starts a process of inflammation (step 3), activation of hepatic stellate cells leading to fibrosis and then cirrhosis, which involve secretion and progressive accumulation of ECM components (step 4).

Our results reveal ASGPR sH2a as a novel non-invasive marker for liver fibrosis and function. SH2a, the enzymes ALT, AST, GGT and ECM components like collagen and hyaluronic acid (HA) reflect different stages of the fibrogenic process. Hepatocytes are targets for most insults to the liver, including hepatitis viruses and alcohol [16]. The damage caused abrogates the function of the hepatocytes and leads to subsequent release of inflammatory factors that activate hepatic stellate cells and cause them to differentiate to myofibroblasts that secrete ECM components and matrix metalloproteinases that degrade them [17], [18]. The secreted ECM components and their degradation products accumulate, originating fibrosis that further impairs liver function and develops to cirrhosis (Fig. 3). Thus, in the chain of events that leads to fibrogenesis, HA is a late indicator of the production of ECM; the enzymes are released by damaged cells; whereas we suggest that sH2a is a marker of liver function, correlating to the mass of functional hepatocytes (Fig. 3). Reduction of the levels of sH2a would reflect early events in the fibrogenic process, affecting hepatocyte function. We can speculate that the few cases where sH2a levels were abnormally high might indicate the start of a neoplastic process with proliferation of hepatocytes that express high levels of the ASGPR. At later tumorigenic stages, with dedifferentiation, one would expect again very low levels of sH2a. This is a subject that requires further studies. On the other hand, during the development of fibrosis, temporary changes in the total hepatocyte function (indicated by sH2a levels) may not occur simultaneously with changes in the release and clearance of damage markers (e.g. ALT). The function (sH2a) and damage markers give complementary information and when combined provide an accurate prediction of the fibrosis stage, including early and intermediate stages of fibrosis that other markers fail to detect. In our combined algorithm, the higher weight given to sH2a reduces the influence of ALT increases due to non-hepatic sources. SH2a is liver-specific, but we still do not know whether its blood levels are modulated or not in other diseases, for example in kidney disease, which could be a potential limitation.

HA, as well as other proposed tests, can only differentiate very mild from very severe disease ([14] and our unpublished results). With our sH2a, ALT combination score a cutoff <4 gives a negative predictive value for fibrosis <2 of 92.9% and a positive predictive value for fibrosis > = 2 of 62.1%. This fairs very well even when comparing with the predictive value of other markers for severe disease. For example, in one study a 60 µg/l HA cutoff gave a high negative predictive value (99.5%), for the absence of cirrhosis but very low positive predictive value in predicting cirrhosis (30%) [19]. This was improved by combination of the levels of HA with those of AST and albumin, which gave a good prediction for absence of severe fibrosis but unsatisfactory for intermediate levels [20]. Similarly, a better known combination method, Fibrotest, is useful for detecting advanced fibrosis but not very satisfactory at intermediate levels [14]. It has been argued that biopsy itself has only an 80% accuracy [21]. The simple combination of sH2a and ALT gave an AUROC of 0.786 for intermediate fibrosis (stages 2–3), which is better than other markers or combinations of markers. For advanced fibrosis and cirrhosis (stages 3–4) the AUROC obtained was 0.863, which compares very favorably with the AUROC obtained for severe disease with combinations of other markers, e.g. AUROC = 0.76 for significant fibrosis and AUROC = 0.82 for cirrhosis in an AST to platelet ratio [22], AUROC = 0.81 for cirrhosis with combined HA, TIMP-1, and platelet count [23] and AUROC = 0.84 with Fibrotest [21].

A method that is being increasingly used is transient elastography (FibroScan), which measures liver stiffness, which in turn is correlated to fibrosis stage. Using this method the AUROC values obtained for significant fibrosis ranged from 0.76 to 0.83, quite suitable but lower than with the combination of sH2a with ALT [24], [25]. Like in other methods, the values that transient elastography provides for moderate fibrosis are not satisfactory; for example in one recent study it could not statistically differentiate stage 2 from 0–1 [26]. In addition, transient elastography values are inaccurate in obese patients or in those with fatty liver and the method cannot be used in patients with ascites.

The sensitive assessment of hepatocyte function by sH2a could allow the measuring of success of patient treatment. In a longitudinal study during treatment of HCV patients, the change in sH2a levels correlated, unlike other markers, with the success of the therapy (our unpublished results). Studies of membrane ASGPR levels upon resection and recovery or in liver transplantation suggest that sH2a could also be measured to monitor these interventions [27].

SH2a should be further evaluated as it emerges as a good candidate for a suitable liver fibrosis marker. It has been argued that such a marker should: be specific for the liver, be readily available, not be subject to false positive results, identify the stage of fibrosis [13].
